# Using Twitter to Understand the Human Bowel Disease Community: Exploratory Analysis of Key Topics

**DOI:** 10.2196/12610

**Published:** 2019-08-15

**Authors:** Martín Pérez-Pérez, Gael Pérez-Rodríguez, Florentino Fdez-Riverola, Anália Lourenço

**Affiliations:** 1 Department of Computer Science University of Vigo Escuela Superior de Ingeniería Informática Ourense Spain; 2 Biomedical Research Centre Campus Universitario Lagoas-Marcosende Vigo Spain; 3 Next Generation Computer Systems Group, School of Computer Engineering Galicia Sur Health Research Institute Galician Health Service - University of Vigo Vigo Spain; 4 Centre of Biological Engineering Campus de Gualtar University of Minho Braga Portugal

**Keywords:** inflammatory bowel diseases, irritable bowel syndrome, social media, communication, data mining, natural language processing, infodemiology

## Abstract

**Background:**

Nowadays, the use of social media is part of daily life, with more and more people, including governments and health organizations, using at least one platform regularly. Social media enables users to interact among large groups of people that share the same interests and suffer the same afflictions. Notably, these channels promote the ability to find and share information about health and medical conditions.

**Objective:**

This study aimed to characterize the bowel disease (BD) community on Twitter, in particular how patients understand, discuss, feel, and react to the condition. The main questions were as follows: Which are the main communities and most influential users?; Where are the main content providers from?; What are the key biomedical and scientific topics under discussion? How are topics interrelated in patient communications?; How do external events influence user activity?; What kind of external sources of information are being promoted?

**Methods:**

To answer these questions, a dataset of tweets containing terms related to BD conditions was collected from February to August 2018, accounting for a total of 24,634 tweets from 13,295 different users. Tweet preprocessing entailed the extraction of textual contents, hyperlinks, hashtags, time, location, and user information. Missing and incomplete information about the user profiles was completed using different analysis techniques. Semantic tweet topic analysis was supported by a lexicon-based entity recognizer. Furthermore, sentiment analysis enabled a closer look into the opinions expressed in the tweets, namely, gaining a deeper understanding of patients’ feelings and experiences.

**Results:**

Health organizations received most of the communication, whereas BD patients and experts in bowel conditions and nutrition were among those tweeting the most. In general, the BD community was mainly discussing symptoms, BD-related diseases, and diet-based treatments. Diarrhea and constipation were the most commonly mentioned symptoms, and cancer, anxiety disorder, depression, and chronic inflammations were frequently part of BD-related tweets. Most patient tweets discussed the bad side of BD conditions and other related conditions, namely, depression, diarrhea, and fibromyalgia. In turn, gluten-free diets and probiotic supplements were often mentioned in patient tweets expressing positive emotions. However, for the most part, tweets containing mentions to foods and diets showed a similar distribution of negative and positive sentiments because the effects of certain food components (eg, fiber, iron, and magnesium) were perceived differently, depending on the state of the disease and other personal conditions of the patients. The benefits of medical cannabis for the treatment of different chronic diseases were also highlighted.

**Conclusions:**

This study evidences that Twitter is becoming an influential space for conversation about bowel conditions, namely, patient opinions about associated symptoms and treatments. So, further qualitative and quantitative content analyses hold the potential to support decision making among health-related stakeholders, including the planning of awareness campaigns.

## Introduction

Mass access and diffusion of health-related news and information have dramatically changed in recent years. In addition to traditional media, the internet has become a pivotal instrument for sharing knowledge [1]. In this context, the use of social media has increased exponentially over the last years. More and more people and institutions, including governments and health organizations, use social networks, blogs, content-sharing sites, and wikis on a regular basis [2-4]. Therefore, social media have become a major source of information [5]. Social media creates the opportunity for users to interact among large groups of people that share the same interests and suffer the same afflictions. In particular, these channels promote the ability to find and share information about health and medical conditions [6,7]. Notably, many people who suffer from chronic diseases resort to groups or communities in social networks to share experiences and stand by for news about their afflictions [8-10]. One recurrent example is inflammatory bowel disease (IBD), a chronic, relapsing, and remitting autoimmune disorder with 2 main conditions, Crohn disease and ulcerative colitis [11]. The worldwide incidence of these conditions has been increasing over the last few decades [12]. IBD is frequently diagnosed in the second to fourth decades of life, with a high incidence during the peak female reproductive years [13]. Communities all around the world elected May 19 as the World IBD Day, a reference date to raise awareness of this chronic disease and its associated symptoms [14].

Nowadays, there is an increasing interest in analyzing and studying several factors related to health topics in social media, especially on Twitter [15-17]. Namely, text mining (TM) and natural language processing methods and techniques are being applied to systematically identify the topics under discussion as well as study the relationships among individuals and topics. Indeed, over recent years, an increasing number of publications reported health-related studies in social media. For example, how promotional health information about Lynch syndrome impacts laypeople’s discussions [18]; diabetes-related participation on Twitter by describing the frequency and timing of diabetes-related tweets, the geography of tweets, and the types of participants [19]; understanding the use of social media by patients with different types of cancer [17]; and the emergence of health online communities of practice (ie, group of people who share experiences) [16].

Regarding IBD, only a few studies exist, and these are focused on the analysis of particular user communities or particular user details. For example, the study by Guo et al studied the use and quality of social media in patients with IBD [20], and the study by Keller et al discussed how individuals taking IBD medication during reproductive periods made decisions about their medication use [21]. So, the aim of this study was to gain a better understanding of the communities involved in the dissemination of information about bowel disease (BD). A collection of over 24,000 tweets related to BD enabled the identification and geolocation of active users and the analysis of external events that determine tweet content and discussion, as well as the relations between the biomedical and scientific topics being mentioned. The obtained results are useful to understand the most relevant topics and communities and, specifically, how patients discuss, feel, and react to symptoms, changes in habits, and medication. That is, to learn how to enhance the dissemination of information and raise awareness among patients, which are the 2 main objectives of health-related stakeholders.

## Methods

### Twitter Communication Environment

Currently, the architecture of Twitter supports different user actions in response to a tweet (ie, replies, retweets, and favorites), each of them holding a specific meaning in terms of communication capabilities. Notably, replies represent the specific response to a sent tweet, retweets stand for the reposting of tweets (which is useful to quickly share and promote content), and favorites indicate that the content of a tweet is highly appreciated by the community and can be seen as a user tweet bookmark.

Figure 1 exemplifies the aforementioned communication modes. On the one hand, replies and retweets (ie, user relations) allow identifying users who support or discuss any sent message. For example, users B and C are interested in a tweet published by user A. This allows to identify how the information is spread and which are the most influential users. On the other hand, the number of retweets and favorites (ie, tweet interactions) helps to measure the relevance of the new content to the community. For example, user D likes the original tweet sent by user A and saves it for future reference.

### General Workflow

Figure 2 depicts the workflow implemented in this study to retrieve, process, and analyze BD-related tweets, which consisted of 2 fundamental phases: (1) data collection and filtering and (2) corpus processing and analysis.

From February 1, 2018, to August 31, 2018, tweet data were retrieved via the Twitter application programming interface (API). Tweet contents and associated information were processed: whenever possible, users were geolocated, their gender was determined, and they were identified as organization or patient; tweet contents were cleaned (hashtags and mentions to user accounts were removed) and prepared for further text processing; and, entity recognition and sentiment analysis were applied.

### Data Collection and Filtering

Data collection accounted for tweets containing terms as *Inflammatory bowel disease*, *Irritable bowel disease*, *Irritable colon*, *Ulcerative colitis*, *Ileocolitis*, *Ileitis*, *Crohn*, *Granulomatous*, and *Jejunoileitis*. The Java library Twitter4J [22] was used to perform such collection. From all the retrieved tweets, only those written in English were considered to ensure the consistency of further examination. A total of 4.10% (1055/25,689) tweets written in other languages, such as French, Spanish, or Italian, were eliminated. The final dataset comprised 24,634 unique tweets written in English by 13,295 different users.

### Corpus Processing and Analysis: User Characterization

All the tweets were automatically labeled by tweet creator. Data present in user profiles were collected and further validated (see details in the next subsections). Specifically, user characterization involved gender determination, differentiation of organizations and patients, and geolocation.

Unfortunately, it was not possible to determine the age of the users because of the low precision of current prediction models and because several studies point that most Twitter users fall into a small range of years [23].

#### Face and Gender Recognition

Gender identification entailed a 2-step strategy based on a gender-name dictionary [24] and a convolutional neural network model (this model was trained over more than 500,000 public face images extracted from IMDb and Wikipedia and was reported to have nearly a 90% of accuracy) [25]. First, the user name was checked against the dictionary. If there was a perfect match, that is, a unique gender associated with the name, the gender was resolved. Otherwise, and if there was a user profile picture, the deep learning model was applied. If there was no user profile picture, or the model could not recognize a single face in the image, the gender was set as unknown.

#### User Identification

Whenever possible, user accounts were categorized as belonging to organization, individual (ie, patient and medical expert), and unknown. The strategy to identify users was focused on the analysis of the user account (ie, name and description).

First, to identify users as organizations, several cascade steps were followed, namely, (1) the account had a country, country code, or a continent in the user name; (2) the account had a URL domain in the name (eg, .org); and (3) usage of regular expressions to check for nonpersonal keywords in the description (eg, official, news, info, or pharma).

If the user was not identified as an organization, the following steps were applied to check if the user was an individual: (1) the account had a recognized gender; (2) the account was recognized by Twitter as a contributor or a translator; (3) the description was written using first-person pronouns and their variant forms (ie, possessive and reflexive); (4) the description had emojis or emoticons; and (5) usage of regular expression to check for person abbreviations (eg, Ms or Mr).

Finally, to differentiate BD experts (eg, doctors, medical staff, or researchers) from patients, all accounts identified as individuals were processed with an additional recognition step to check for expert-related keywords (eg, Dr, Prof, MD, or PhD).

Whenever the strategy could not help determine the type, the user was labeled as an unknown type.

#### Geolocation

Twitter does not require users to specify the location. When users introduce such data, it is in the form of free text, which often raises consistency issues in further analysis (eg, a user can enter *NYC* and others may identify the same location as *New York City*). Another issue to take into account is the existence of cities in different countries that share the same name (eg, *Guadalajara* is a city both in Spain and Mexico).

Thus, the applied location identification method took into consideration information about the time zone, the Coordinated Universal Time (UTC) offset, and the location text. These data were used in combination with the GeoNames database [26], which contains over 10 million geographical names and is accessible through a free Web service. The data extracted from Twitter were searched against the GeoNames database. If the data were not accurate enough, that is, matching multiple database entries, the time zone and the UTC offset were used to help resolve the location. In those cases where different cities shared the same name and time zone but were located in different countries, the location was set as unknown.

### Corpus Processing and Analysis: Tweet Characterization

To analyze the content of the generated corpus, it was essential to be able to properly recognize the relevant (topic related) terms mentioned in the tweets. For this purpose, several text preprocessing techniques were applied and then an in-house–developed named entity recognizer supported the annotation of terms pertaining to the selected BD-related semantic categories.

#### Data Cleaning

As a first step, the following preprocessing tasks were applied to the content of the tweets in the dataset:

- Removal of special characters that did not provide useful information (eg, *&*, *(“, “)*, *, *+*, *<*, or *>*)
- Identification of replies and mentions to other users (represented with *@*) and extraction of URLs
- Removal of the symbol *#* in hashtags, and split of hashtags in multiple words (if possible) with the goal of revealing relevant terms to the analysis (eg, *InflammatoryBowelDisease* to *Inflammatory Bowel Disease)*. All these operations were carried out using the Twitter text library [27]
- Deletion of repeated letters if there are 3, or more, consecutive and identical characters (eg, *haaaapppyy* to *haappyy*)
- Correction of spelling errors using the Hunspell dictionary [28], a collection of specific medical terms [29] obtained from the OpenMedSpel [30], and the MTH-Med-Spel-Check tool [31]. The correction was done automatically by selecting the suggested word with the highest similarity with the original (incorrect) term. The similarity was calculated using the normalized Levenshtein algorithm [32]
- Expansion of abbreviations and shorthand terms, which were not included in the Hunspell dictionary (eg, *SBBOS* to *small bowel bacterial overgrowth syndrome*). Although Twitter has increased the maximum length of tweets from 140 to 280 characters, the use of abbreviations is still very common. Therefore, a custom dictionary of abbreviations was constructed in house, comprising terms extracted from multiple locations [33-35].

Then, a new round of text preprocessing tasks prepared the tweets for named entity recognition, namely, tokenization (ie, breaking a stream of text up into words, phrases, or other meaningful elements), stop word removal (ie, removal of too frequent, not content-bearing tokens), part of speech tagging (ie, to assign a lexical category to each token), and lemmatization (ie, to obtain the lexeme form of the token). Beside single word tokens (unigrams), bigrams and trigrams, that is, contiguous of 2 or 3 sequences of tokens, were also considered in entity recognition. All the aforementioned tasks were implemented using the Stanford CoreNLP pipeline [36].

#### Named Entity Recognition

The semantic lexicon applied in named entity recognition was mostly retrieved from the repository of biomedical ontologies BioPortal [37] as follows: the Human Disease Ontology (DOID) [38], which provides descriptions of human disease terms, phenotype characteristics, and related medical vocabulary; the Ontology For Nutritional Studies (FoodOn) [39], which covers human food raw ingredients, food products, and product types and develops semantics to food production, culinary, nutritional, and chemical ingredients and processes; the Symptom Ontology (SYMP) [40], which covers disease symptoms, with symptoms encompassing perceived changes in function, sensations, or appearance reported by a patient indicative of a disease; and the branch *Intervention or Procedure* of the National Cancer Institute Thesaurus (NCIT) [41], which describes treatments or actions taken to prevent or treat disease or improve health in other ways. The DrugBank ontology supported the recognition of chemical, pharmacological, and pharmaceutical terminology, that is, approved small molecule drugs, approved biotech (protein and peptide) drugs, nutraceuticals, and experimental drugs [42].

As a whole, the lexicon supporting the entity recognition encompassed a total of 217,468 term entries. For the sake of simplicity, the results of the semantic annotations are presented and discussed in terms of the *meta* categories, that is, *Drug* encloses all the classes encompassed by DrugBank, *Food and Diet* refers to the food ingredients and food products classified by FoodOn, *Symptom* relates to the symptoms as presented by SYMP, *Treatment* refers to the treatments classified by NCIT, and *Disease* groups together disease terms, phenotype characteristics, and related medical vocabulary, as described by DOID.

The named entity recognition pipeline was implemented in house and entailed dictionary lookup, as well as pattern- and rule-based recognition. To be able to match the lexicon with tweet contents, the lexicon required some processing, namely, convert all terms to lowercase, remove extra whitespaces, remove small and long terms (ie, less than 2 characters and terms longer than the maximum tweet length), replace special characters by a whitespace, and remove terms associated with more than one category.

An inverted recognition technique was used in actual entity recognition [43]. This technique uses the words in the text as patterns to be matched against the lexicon. This was a valid approximation for this study because the number of words in the tweets were much smaller than the number of terms in the lexicon, that is, a fewer number of patterns to match. Recognition preference was given to the longest possible n-grams. In addition, the recognizer accepted perfect matches as well as lexical variations of the terms (ie, lemmatized entries and abbreviations).

#### Sentiment Analysis

The sentiment of the tweets was analyzed using the Valence Aware Dictionary and sEntiment Reasoner (VADER) API for Python [44]. VADER is a lexicon- and rule-based sentiment analysis tool that is specifically attuned to sentiments expressed in social media. The predicted sentiment (ie, compound score) is computed by summing the valence scores of each word in the lexicon, adjusted according to emotion-related rules, and then normalized to have values between −1 (most extreme negative emotion) and +1 (most extreme positive emotion).

#### Network Representation and Analysis

Graph analysis was performed to measure the relevance of individual terms as well as term-term pairs. Specifically, this analysis was applied to user interactions, via mentions and retweets (ie, directed mentions and retweets), and co-occurrence of semantically meaningful terms (ie, whenever 2 terms were found in the same tweet, these 2 terms were considered to share a link).

Networks were generally described in terms of the number of nodes and edges, as well as metrics of degree, characteristic path length, clustering coefficient, and the average number of neighbors [45]. In more detail, graph connectedness was measured by degree centrality, betweenness centrality, and closeness centrality [46-48]. Briefly, degree centrality measured the total amount of direct links with the other nodes (ie, higher degree implies the node is more central), betweenness centrality measured the *mediation* role of the nodes (ie, if other nodes have to go through the node to ensure communication, then the node is likely important and has a high betweenness centrality), and closeness centrality measured the convenience and ease of connections between each node and the rest of the nodes (ie, if the average shortest path of the node is small, then the node has a high closeness centrality) [49,50].

The clustering coefficient was used to measure the degree to which nodes tend to cluster together. Evidence suggests that social network nodes tend to create tight groups, which are characterized by a relatively high density of links; this likelihood tends to be greater than the average probability of a link randomly established between 2 nodes [51,52].

## Results

### Overview

The Results section was structured following the logical order of the proposed questions. Figure 3 illustrates how particular research questions were answered by a certain analysis and identifies the main insights provided by each of these analyses. Most notably, results were structured such that the basic characterization of the BD-related tweets in terms of user activity and topics of interest were presented first and, then, the interrelation of topics in the conversations was detailed along with the visibility of external sources of information.

The ability to identify the most active users (ie, users that post more tweets) and topic-specific communities (eg, gluten-centered community) is pivotal to gain a better understanding about how to adapt or finetune the communication (ie, reach a broader audience or focus on a specific community) as well as discover influencers (ie, *vessels* of information distribution, within or across the communities). The analysis of user activity (different time windows may apply to different communities) is interesting as a means to plan the best time to post a new tweet (ie, when to expect major user attention).

The semantic annotation of tweet contents goes a step forward, providing knowledge about the topics being discussed (ie, individually and in combination). Likewise, the identification of tweets linking to external information sources is relevant to bring forward the visibility these sources are receiving through Twitter.

A health-related stakeholder is a typical example of someone that can benefit from the insights provided by the overall study. Say that the aim is to plan an awareness campaign about BD and food habits. Likely, this stakeholder wants to study the target audience in terms of topics that are attracting more attention and tweeting habits. The campaign may be planned to receive short-term attention (eg, announcing the launch of a novel drug or a new food supplement) or promote awareness throughout a longer period of time (eg, promote healthier food habits).

### Bowel Disease Communities

User relations, that is, mentions and retweets, were represented in a network to study communication interplay, namely, to identify target communities and influential users (ie, individuals and/or institutions with high audiences). Figure 4 depicts this network such that the nodes denote the users (ie, account name), the node size is based on the node in-degree (ie, the users that received most communication are represented by bigger nodes), and the edges account for the number of mentions and retweets. The 4 different background areas in the figure denote the communities, mostly characterized by account descriptions: gluten-, nutritional-, BD-, and food-related relations. In this study, only communities with more than 5 connected users were considered.

In this network, the out-degree of the nodes (ie, users initiating communication with other users) is much lower than the in-degree of the nodes (ie, users receiving significant tweets). The user accounts @CrohnsColitisUK, @CrohnsColitisFn, and @HealioGastro, which belong to disease-specific organizations and have primarily informative/educational goals, were among the accounts receiving more communication (ie, higher in-degree). This conveys the rationale that individuals typically prefer to ask for health information to trusted organizations [6,53,54]. Conversely, accounts of BD patients (eg, @colitisandme) and experts in bowel conditions or nutrition (eg, @IBDMD and @charlie_lees) were among those showing highest out-degree.

Public lists of BD-related influencers [55] supported the identification of organization accounts, such as @CrohnsColitisUK, @HealioGastro, and @ACCUCatalunya, and personal accounts, such as @colitisandme, @IBDMD, and @EdwardLoftus2, that are typically reached for medical advice.

Figure 5 shows an example of a tweet exchange: the user @Crohnoid, a Crohn disease patient, asks the user @ibddoctor, an expert in BD, about 2 possible diagnostic techniques, that is, magnetic resonance imaging and computed tomography; @ibddoctor explained the advantages and disadvantages of these techniques, and another user @SandraZelinsky also intervened, pointing out limited access.

### Demographic Distribution and Hot Zones

Knowledge about how the community is demographically distributed is important to carry out public information campaigns and study the impact of government and institutional actions in different demographic areas. In this analysis, 59.98% of the users (7975/13,295) were geographically distributed all around the world.

Figure 6 shows the geographical distribution of the BD communities. The size of the nodes represents the number of users located in each country (ie, the bigger the circle, the higher is the number of users) and colors identify the continents (ie, blue for America, purple for Europe, brown for Africa, green for Asia, and red for Australia).

In general, most of the users were located in the United States, the United Kingdom, Canada, and Australia, which is consistent with current knowledge about the prevalence of these conditions [56]. The highest reported prevalence is in Europe (with the highest prevalence of ulcerative colitis in Norway and of Crohn disease in Germany) and North America (with the highest prevalence of ulcerative colitis in the United States and Crohn disease in Canada). The prevalence of IBD exceeded 0.3% in North America, Oceania, and many countries in Europe.

The number of users from India, Bangladesh, and the Philippines was also noticeable and may be explained by the rising incidence of BD conditions in newly industrialized countries in Africa, Asia, and South America.

### Biomedical and Scientific Topics

Understanding what the user community is talking about was important to identify the topics that received more attention and to be able to better align new information/communication strategies with the interests of the community. Table 1 presents the top 25 most mentioned terms along with their corresponding semantic category (ie, *Food and Diet*, *Disease*, *Treatment*, *Symptom*, and *Drug*) and sorted by the number of including tweets. Explicit mentions to IBS and IBD (eg, *Inflammatory Bowel Disease* or *Ulcerative Colitis*) and noncontent–bearing, generalist terms (eg, *Disease* or *Food*), were at the top of term mentions but were not listed in the table.

The #Tweets column indicates the number of tweets in which a term is mentioned, the #Favorites column indicates the number of times that a tweet containing a term was selected as a favorite, the #Retweets column indicates the number of times that a tweet containing a term was retweeted, and the #Hashtags column indicates the number of times that a term was used in hashtags, including term variations (eg, *anxiety disorder* like *#anxiety*).

The volume of retweets is useful to understand how the information flows among users, whereas the number of favorites can be understood as a metric to measure the usefulness of the tweets containing the term [57,58]. In addition, the number of times a term appears in hashtags is interesting to understand how the user wishes the tweet to be indexed (to be easily found by others with similar interests as well as to bring attention to certain topics). This can be understood as a metric to measure the impact of the term in the community [59].

As illustrated in Table 1 (and in the topological analysis of the co-occurrence network of terms in Multimedia Appendix 1), the most mentioned terms related to *Disease* (35.64%, 11,688/32,794) and *Food and diet* (25.43%, 8342/32,794) categories, followed by terms from *Symptom* (17.71%, 5811/32,794), *Treatment* (14.83%, 4864/32,794), and *Drug* (6.37%, 2089/32,794) categories, respectively.

Diarrhea (10.33%, 1208/11,688 of the mentions to disease terms) and *constipation* (8.94%, 1046/11,688 of the mentions to disease terms) were the most mentioned disease-related terms. This was somewhat expected considering that many IBD and IBS patients have diarrhea as a side effect of the disease and many others claim to have problems of constipation [60]. Interestingly, constipation is one of the terms most often included in hashtags, which indicates that the topic is meaningful and timely to the community. Other diseases typically associated with BD conditions were also discussed, notably *cancer* (3.37%, 395/11,688 of the mentions to disease terms), *anxiety disorder* (3.04%, 356/11,688 of the mentions to disease terms), *depression* (2.49%, 292/11,688 of the mentions to disease terms), *arthritis* (1.71%, 200/11,688 of the mentions to disease terms), and *asthma* (1.46%, 171/11,688 of the mentions to disease terms). Chronic inflammation is known to be a major risk factor for the development of gastrointestinal malignancies and is often associated with inflammatory arthritis. Notably, as the population of patients with IBD grows older, with longer periods of chronic inflammation and longer exposure to immunosuppression, there is an increased risk of developing cancer [61] and arthritis [62]. Moreover, studies show that the rates of anxiety and depression tend to be higher among patients with Crohn disease or ulcerative colitis compared with those with other diseases as well as the general population [63,64]. Finally, interest in discussing asthma is justified by the association between this disease and early- and late-onset ulcerative colitis, particularly because of shared environmental risk factors [65]. Posts discussing BD and other diseases often show high retweet rates, and it is noticeable that depression is currently at the center of attention (ie, those tweets have the greatest number of favorites).

Symptoms typically associated with the different stages of the disease such as *bloating* (4.40%, 256/5811 of the mentions to symptom terms), *flatulence* (3.20%, 186/5811 of the mentions to symptom terms), and *abdominal pain* (2.97%, 173/5811 of the mentions to symptom terms) were also discussed with considerable frequency and the containing tweets were among the most retweeted, that is, people within this community find it relevant to broadcast information related with the symptomatology of BD conditions (eg, symptom-disease evidence and symptom relief therapies) [60].

The BD community is also sharing information about diet-based treatments and shows particular interest in *gluten-free dietary interventions* [66] and *probiotics* [67] (both with a 7.32%, 611/8342 and 544/8342 of the mentions to food and diet terms). Although the characteristics of gluten sensitivity in IBD remain unclear, gluten is known to generate peptides that can alter intestinal permeability and affect the immune system [68]. Recent studies investigated the effects of a wheat gluten–containing diet on the evolution of sodium dextran sulfate–induced colitis [69] and evaluated the usefulness of a low fermentable oligosaccharides, disaccharides, monosaccharides, and polyols diet on patients with IBS, nonactive IBD, and celiac disease compared with a gluten-free diet [70]. Likewise, some prebiotics, such as germinated barley foodstuff, psyllium, or oligofructose-enriched inulin, might provide some benefit in patients with active ulcerative colitis or ulcerative colitis in remission [71]. Other studies suggest that the VSL#3 probiotic may be effective in inducing remission in active ulcerative colitis [72]. The high number of favorites, retweets, and hashtags reflect the interest of the BD community in knowing more about these dietary interventions and discuss the pros and cons of different diets and foods.

**Table 1:** Top 25 mentioned terms sorted by the number of tweets.

Term	Category	Number of tweets	Number of favorites	Number of retweets	Number of hashtags
Diarrhea	Disease	1009	487	271	45
Constipation	Disease	971	359	213	152
Pain	Symptom	701	682	267	112
Medical cannabis	Drug	630	1134	614	100
Gluten	Food and Diet	519	1952	191	62
Probiotic	Food and Diet	498	936	344	142
Celiac disease	Disease	376	542	221	16
Cancer	Disease	367	691	424	50
Anxiety disorder	Disease	338	724	269	52
Depression	Disease	283	1107	501	51
Bloating	Symptom	264	158	80	52
Fibromyalgia	Disease	213	362	161	74
Dietary supplement	Food and Diet	196	602	146	9
Arthritis	Disease	190	872	375	32
Myocardial infarction	Disease	188	122	77	17
Allergic hypersensitivity disease	Disease	179	471	241	22
Flatulence	Symptom	175	482	35	6
Abdominal pain	Symptom	172	801	129	6
Multiple sclerosis	Disease	172	480	243	15
Asthma	Disease	170	486	259	12
Obesity	Disease	152	370	186	24
Vitamin D	Food and Diet	148	96	49	26
Heart disease	Disease	148	289	142	33
Autistic disorder	Disease	144	435	210	29
Hypnotherapy	Treatment	131	210	131	29

In general, drugs and nondiet-based treatments are less mentioned than the other categories. However, *medical cannabis* (36.38%, 760/2089 of the mentions to drug terms) and *hypnotherapy* (3.57%, 174/4864 of the mentions to treatment terms) raise some attention as an alternative, nonconventional treatments. Although these results are still inconclusive, recent studies show improvements in some BD-related symptoms. Medical cannabis is being tested for the treatment of gastrointestinal disorders such as abdominal pain and diarrhea. Experimental tests show that single ingredients from cannabis, such as tetrahydrocannabinol and cannabidiol, are responsible for these effects [73,74]. Conversely, the use of hypnotherapy is being validated in the treatment of gastrointestinal symptoms such as reducing fasting distal colonic motility or reducing systemic and rectal mucosal inflammatory responses [75]. Although, the results of using these alternative therapies remain unclear. As it stands, users show greater interest for cannabis-related posts, as denoted by the high number of tweet favorites and retweets as well as the inclusion of hashtags. In addition, users find these tweets interesting as they choose to disseminate them among their followers and make the topic indexation easier.

### Topics of Patient Communications

A closer look into the tweets posted by BD patients was relevant to better understand how patients feel about and deal with BD symptoms, associated diseases, changes in habits, and medication. This analysis considered only the tweets posted by user accounts identified as patients (58.63%, 7795/13,295 of the total of user accounts). Moreover, it was possible to identify 2800 females (35.92%, 2800/7795 of the user accounts identified as patients) and 3860 males (49.51%, 3860/7795 of the user accounts identified as patients). Typically, the 11,098 tweets (45.05%, 11,098/24,634 of the total of tweets) posted by patients expressed a negative sentiment (51.99%, 5770/11,098), but there were also positive tweets (31.99%, 3551/11,098) and some tweets with a neutral sentiment (13.99%, 1553/11,098).

The high amount of negative opinions is explained by the fact that patients are known to use social platforms to *vent out* their emotions, namely, their frustrations when it comes to diseases that do not have a cure, such as in the case of IBD [76,77]. Table 2 describes the tweets of patients in terms of the recognized semantics terms and the tweet sentiment.

**Table 2:** Distribution of the sentiment of tweets by semantic category.

Category	Negative tweets, n (%)	Neutral tweets, n (%)	Positive tweets, n (%)	Total number of tweets, N
Disease	5691 (54.00)	1686 (16.00)	3161 (30.00)	10,538
Symptom	1152 (67.02)	103 (5.99)	464 (26.99)	1719
Food and Diet	1303 (47.01)	222 (8.01)	1247 (44.98)	2772
Drug	237 (36.02)	118 (17.93)	303 (46.05)	658
Treatment	710 (43.99)	339 (21.00)	565 (35.01)	1614

An important part of the tweets expressing positive emotions was related to gluten-free diets and probiotic supplements. In this line, there were more positive tweets posted by females than males (57.98%, 2274/3922 of the female tweets against 51.99%, 2390/4597 of the male tweets). However, in general, the tweets containing mentions to foods and diets (Figure 7) showed a similar distribution in terms of negative and positive sentiments. The main reason was that the effects of certain food components (eg, fiber, iron, and magnesium) were perceived differently, depending on the state of the disease and other personal conditions of the patient.

Disease and symptom (Figure 7) were the semantic categories with the highest number of mentions in negative tweets. These tweets discussed the *bad side* of BD conditions, that is, symptoms such as pain, fatigue, and migraines along with the co-occurrence of other conditions, namely, depression, diarrhea, and fibromyalgia [64,78]. In contrast, several tweets containing mentions of drugs (Figure 7) showed positive emotions, namely, the tweets highlighting the benefits of medical cannabis for the treatment of different chronic diseases.

As a means to look into these tweets from another perspective, Figure 8 depicts a subgraph of the semantic co-occurrence network reconstructed from the patient tweets (see details on the topological analysis of the complete network in Multimedia Appendix 2). This subgraph shows the relations between diseases and drugs. The size of the nodes is based on the node degree (ie, bigger nodes represent the terms mentioned in more tweets), whereas the edge size expresses the strength of co-occurrence (ie, thicker edges represent a higher number of term-term occurrences). Red nodes represent drugs and yellow nodes represent diseases, whereas the edge color stands for the tweet sentiment (ie, red indicates a majority of negative tweets, green indicates a majority of negative tweets, and black indicates neutral sentiment).

Although medical cannabis (and its components such as cannabidiol) was the most discussed drug, it was also possible to track down patient discussions about commercial drugs such as l-glutamine, lactulose, loperamide, and *Plantago* seed. For example, patients reported the positive effect of l-glutamine on diarrhea and muscular atrophy, but they also expressed their concern with glutamate leading to the occurrence of anxiety disorders. Glutamine is used to protect the mucous membrane of the esophagus and intestines and can boost immune cell activity in the gut [79,80]. Conversely, glutamine helps to create gamma-aminobutyric acid, a neurotransmitter that can stable the mind but can also produce glutamate, an excitatory neurotransmitter that can overstimulate the brain [81].

Patients discussed the use of laxatives in treating constipation, but not all of the mentioned laxatives were recommended. Notably, lactulose is a sugar that cannot be digested in the gut and thus, tends to cause or aggravate IBS symptoms, such as gas, bloating, discomfort, and cramping [82]. In turn, loperamide was suggested as an effective astringent to treat diarrhea and constipation. Indeed, a previous study reported a significant improvement in stool frequency and consistency [83].

Finally, patients were interested in the beneficial healing properties of medicinal plants such as *Plantago* (in various forms, such as roasted seeds, decoction, or syrup), namely, anti-inflammatory, laxative, and astringent properties [84].

### Temporal Analysis of User Activity

Table 3 describes the number of tweets and the volume of tweet interactions (ie, retweets and favorites) from February 1, 2018, to August 31, 2018. It was interesting to identify the periods of time when users are more likely to tweet and, in particular, how specific events, such as the IBD day, a scientific conference, or an informational campaign, could affect such activity.

In particular, the celebration of the World IBD Day (on May 19, 2018), which is a worldwide event to raise awareness about BD conditions and to urge governments and health care professionals to take action and show support to the sufferers, motivated an increase in tweet interactions, that is, retweets and favorites (ie, 15,820 and 18,158 tweet interactions in May and June 2018, respectively). The average number of retweets during these months increased by 174% (3314/1902) compared with the average number of retweets during the rest of the year. Regarding tweet favorites, the increment was still noticeable (329%, 13,585/4130) compared with the average number of favorites during the rest of the year.

**Table 3:** Monthly activity of posting and interaction during the analyzed period.

Month	Number of tweets, n	Number of tweet interactions, n
February	2930	4285
March	4239	5822
April	3992	6693
May	4202	16,506
June	3422	18,298
July	2366	7955
August	3952	4468

### External Sources of Information

The hyperlinks shared via tweet provided useful information about current health research and development initiatives (public and private), health promotion actions, and other events that are promoted via Twitter as means to reach out to and engage more people. Tables 4 and 5 summarize the results obtained by grouping the URLs into 4 categories: (1) informative or awareness campaigns about BD; (2) scientific sites, articles, and conferences, usually covering new treatments; (3) health Web pages related to a specific disease; and (4) commercial sites. The #Tweets column indicates the number of tweets in which the URL is mentioned, the #Retweets column indicates the number of times that a tweet containing the URL was retweeted, and the #Favorites column indicates the number of times that a tweet containing the URL was selected as a favorite.

Only a small number of external links included in tweets posted by organizations got highly retweeted and labeled as favorite. Notably, the Guts4life Web page (a portal about IBD) got the highest number of retweets and favorites. This sort of analysis can be of aid in identifying the external sources that the community finds most useful/interesting, especially considering that most of them are related to pages describing BD symptomatology and potential treatments.

Regarding the external links included in the tweets shared by patients, their information flow was in the BD community at the same level compared with that of links posted by organizations (an average of 49 retweets against an average of 49 retweets, respectively). In turn, the links shared by patients had a lower average number of favorites compared with those shared by organizations (ie, an average of 44 favorites against an average of 92 favorites, respectively).

Looking into the linked contents, most of the resources were related to posted articles that belonged to highly prestigious journals, namely, *Nature* and *British Medical Journal*, and reported recent research in BD topics (with no particular focus). The presence of commercial links to pages selling *stoner*- and other drug-related products that do not require a medical prescription was also noteworthy.

**Table 4:** Top 10 external sources of information mentioned in the bowel disease tweets posted by organizations. The URLs are sorted by the corresponding sum of the number of tweets, retweets, and favorites.

Source of information	Category	Number of tweets, n	Number of retweets, n	Number of favorites, n
Guts4life [85]	Health Web page	7	164	1918
Are Your Digestion Troubles Irritable Bowel Syndrome? [86]	Informative or campaign	5	128	231
About Crohn’s Disease [87]	Informative or campaign	1	69	63
Can You Treat Irritable Bowel Syndrome with Cannabis? [88]	Informative or campaign	4	34	84
How to Manage Irritable Bowel Syndrome with Your Brain [89]	Informative or campaign	3	30	77
Inflammatory Bowel Disease (IBD) [90]	Health Web page	10	45	51
Why I Get Excited When You Say You Know Someone With IBD [91]	Informative or campaign	3	27	65
New Treatment Options for Inflammatory Bowel Diseases [92]	Scientific	1	30	57
Supporting Someone With IBD: A Guide For Friends and Family [93]	Health Web page	2	31	48
Chronic Inflammation [94]	Informative or campaign	5	48	26

**Table 5:** Top 10 external sources of information mentioned in the bowel disease tweets posted by patients. The URLs are sorted by the sum of the corresponding number of tweets, retweets, and favorites.

Source of information	Category	Number of tweets, n	Number of retweets, n	Number of favorites, n
Ginger for Nausea, Menstrual Cramps and Irritable Bowel Syndrome [95]	Informative or campaign	1	62	122
Symptoms of Ulcerative Colitis [96]	Commercial	1	178	1
Medicinal Marijuana as a Treatment for IBD Inflammatory Bowel Disease [97]	Commercial	8	45	94
A Starbucks barista called 911 [98]	Informative or campaign	1	24	99
Advances in Inflammatory Bowel Disease Pathogenesis: Linking Host Genetics and the Microbiome [99]	Scientific	1	47	70
Fungal Microbiota Dysbiosis in IBD [100]	Scientific	1	47	70
Murine Colitis Reveals A Disease-Associated Bacteriophage Community [101]	Scientific	1	47	70
I Am LOVING These Probiotics! [102]	Informative or campaign	1	83	27
Effects of Prebiotics vs a Diet Low in FODMAPs in Patients With Functional Gut Disorders [103]	Scientific	2	28	56
Acute GI Bleeding [104]	Health Web page	2	31	50

## Discussion

### Principal Findings

The objective of this paper was to characterize and study the BD community on Twitter. To do so, a dataset of tweets related to BD was collected, processed, and analyzed. The dataset covered a consecutive period of 8 months, from February 1, 2018, to August 31, 2018. As a whole, this analysis provided new insights into 6 main questions: Which are the main communities and most influential users?; Where are the main content providers from?; What are the key biomedical and scientific topics under discussion? How are topics interrelated in patient communications?; How do external events influence user activity?; What kind of external sources of information are being promoted?

Health organizations and BD experts (eg, @CrohnsColitisUK and @IBDMD) were the users that received more tweets, typically looking for trusted information about the conditions. Patients shared experiences among themselves or asked for medical advice. Moreover, the most active users were located in the United States and the United Kingdom, which are among the demographic regions with highest BD prevalence.

Most of the tweets talked about BD symptoms, related diseases, foods, and diets. Specifically, diarrhea, constipation, and pain were the symptoms that raised more concern (in general as well as among patients), whereas gluten and probiotics were among the most discussed dietary interventions (including a high number of favorites and retweets). In this line, females showed higher positive emotions about these dietary interventions than males. Medical cannabis was the most commented drug, notably in the tweets that raised the highest number of favorites, and patients actively discussed the beneficial effects of cannabis (and its components) in mitigating common BD symptoms. Regarding more commercial drugs, patients expressed positive emotions with the usage of l-glutamine on diarrhea and muscular atrophy but also reported negative sentiments because of the production of glutamate and its influence on anxiety disorders. Another notable group of drugs in the discussion were the laxatives, for example, the usage of lactulose was associated with negative emotions because it tends to cause or aggravate IBS symptoms, whereas loperamide was noted to be an effective astringent to treat diarrhea and constipation.

Users were more active during and after the World IBD Day, which shows that these types of initiatives are raising public awareness about these diseases as well as indicates that social networks are part of the routine communication of the BD community. The external resources being shared in tweets by organizations aim to draw people’s attention to awareness/informational sites, whereas those shared by patients typically point to more scientific contents (eg, scientific articles on BD) and alternative treatments (such as cannabis).

### Limitations

The most immediate limitation arises from the fact that the capture of raw data was carried out using the free Twitter API, that is, the identification of users that select one tweet as a favorite as well as the number of available tweets is restricted, with no assurance of a random or representative sample [105]. For this reason, it was not possible to perform a more exhaustive analysis of the social BD communities. Thus, a full data retrieval through automated dashboard vendors, or using a paid service of the Twitter API, may provide further insights.

It should also be noted that this study was based on the assumption that the data entered by Twitter users are true. It is not possible to detect if certain data (eg, the user profile picture or the user location) are reliable. This limitation impacts mainly the analysis of the demographic distribution and the conclusions inferred for a particular gender. That being said, the obtained results were in accordance with the common knowledge of these communities.

Language is another aspect of analysis to take into consideration. This study was only focused on tweets written in English. If the applied techniques are extended to support a greater variety of languages, such as Chinese or Spanish, it may provide complementary findings.

Finally, this study was focused on Twitter. However, the set of social networks might be expanded to analyze a richer dataset from a wide variety of sources. Considering other studies in the literature [21,106,107], Facebook and Instagram would be also sources of interest, although public data access is greatly limited.

### Conclusions and Further Research

In this study, tweets related to BD were analyzed to characterize the user community and the exchanged contents. According to the obtained results, it was possible to detect communities and to describe the most discussed topics among these communities. The large and increasing volume of tweets demonstrates that Twitter is becoming a space for online conversation about BD, namely, associated symptoms and alternative treatments. In addition, the location of users indicates that conversations are happening at a global scale and, motivated by this, health-related stakeholders are using the platform to reach out to a larger audience on a daily basis.

In terms of future research, it would be interesting to perform user classification, that is, being able to identify experts, researchers, and companies, as well as patients. Thus, it would be possible to apply different sentiment analysis and TM approaches to the tweets to explore user-specific motivations, questions, and concerns. For example, it would be interesting to discover the opinion of patients about different treatments and specific symptoms.

## Appendix

Multimedia Appendix 1 Topological analysis of the co-occurrence network of terms for all types of users.

Multimedia Appendix 2 Topological analysis of the co-occurrence network of terms for patient users.

## Abbreviations

API: application programming interface
BD: bowel disease
DOID: Human Disease Ontology
IBD: inflammatory bowel disease
NCIT: National Cancer Institute Thesaurus
TM: text mining
UTC: Coordinated Universal Time
VADER: Valence Aware Dictionary and sEntiment Reasoner
