# Supplementary material for: Using Twitter to Understand the Human Bowel Disease Community: Exploratory Analysis of Key Topics
Source: J Med Internet Res. 2019 Aug 15;21(8):e12610. doi: 10.2196/12610 (PMC6711036; doi:10.2196/12610)
Supplement: Multimedia Appendix 1 [file jmir_v21i8e12610_app1.pdf]

Multimedia Appendix 1

Using Twitter to understand the human bowel disease community: an exploratory analysis of key topics

Martín Pérez-Pérez[1,3,4], Gael Pérez-Rodríguez[1,3,4], Florentino Fdez-Riverola[1,3,4], Anália Lourenço[1,2,3,4*]

[1]The Biomedical Research Centre, Campus Universitario Lagoas-Marcosende, 36310 Vigo, Spain

[2]Centre of Biological Engineering, University of Minho, Campus de Gualtar, 4710-057 Braga, Portugal

[3]Department of Computer Science, University of Vigo, School of Computer Engineering, Campus As Lagoas, 32004 Ourense, Spain

[4]Next Generation Computer Systems Group, Galicia Sur Health Research Institute, Galician Health Service - University of Vigo, Spain

Email addresses:

MPP: martiperez@uvigo.es

GPR: gaeperez@uvigo.es

FFR: riverola@uvigo.es

AL: analia@uvigo.es

[*] Corresponding author:

Anália Lourenço [Tlf.: +34 988 387013, Fax: +34 988 387001]
ESEI: Escuela Superior de Ingeniería Informática. Edificio Politécnico. Campus Universitario As Lagoas s/n, 32004 – Ourense – Spain

Multimedia Appendix 1

Figure 1 illustrates the term co-occurrence network that was constructed to better understand what was being communicated in BD-related tweets. The network was structured using the Circle Pack layout in order to keep the nodes with the same semantic category together [1]. The size of the nodes is based on the node degree (i.e. bigger nodes represent terms that are mentioned in more tweets), while the edge size is calculated based on the strength of co-occurrence (i.e. thicker edges represent higher term-term occurrences). The node color represents the semantic category of the term, i.e., blue stands for treatments, red represents drugs, yellow indicates diseases, green stands for foods and orange represents symptoms.

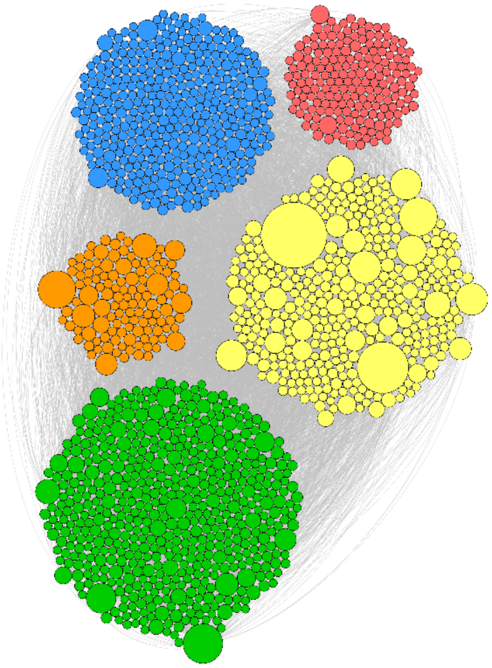

Figure 1. Network representing the co-occurrence of terms in bowel disease-related tweets. The size of the nodes is based on the number of tweets mentioning the term while node color represents the corresponding semantic category.

The complete network has 1,624 nodes and 10,256 edges. Note that, explicit mentions to IBS and IBD (e.g. 'Inflammatory Bowel Disease' or 'Ulcerative Colitis') and non-content bearing, generalist terms (e.g. 'Disease' or 'Food'), were at the top of term mentions (i.e. highest degree values) but were not considered in the analysis. Moreover, it has an average clustering coefficient of 0.555, which expresses to what extent "every term co-occurs with every other term". In the present study, only a few terms were recurrently mentioned in the tweets and so, the clustering coefficient of most of the terms was zero or below ~0.3.

Regarding network connectedness, the degree metric showed the basic connectivity of the term, i.e. the number of other terms it co-occurs with. Naturally, terms referring to the BD-related conditions, such as "diarrhea" (i.e. 450 neighbours), "constipation" (i.e. 338 neighbours), "cancer" (i.e. 236 neighbours), "depression" (i.e. 190 neighbours) and "anxiety disorder" (i.e. 187 neighbours) were among the top most connected nodes.

Regarding the other semantic categories, the most referred terms were as follows: “pain” (i.e. 230 neighbours), “flatulence” (i.e. 130 neighbours), and “bloating” (i.e. 117 neighbours) in symptoms; “diet” (i.e. 60 neighbours), “colonoscopy” (i.e. 51 neighbours), and “meditation therapy” (i.e. 46 neighbours) in treatments; “medical cannabis” (i.e. 80 neighbours), “diamorphine” (i.e. 80 neighbours) and “kaopectate” (i.e. 24 neighbours) in drugs; and, “gluten” (i.e. 245 neighbours), “probiotic” (i.e. 170 neighbours), and “sugar” (i.e. 129 neighbours) in foods.

The terms with highest between centrality were “diarrhea” and “constipation” (i.e. with a value of 0.19 and 0.11, respectively). The third term with higher between centrality was “gluten”, but with a lower value (i.e. a value of 0.07). Considering that this metric shows which nodes act as bridges in network communication, the values showed that it is likely the case that mentions to the two diseases are central in a moderate number of communications.

The observation of closeness centrality values showed “diarrhea” and “constipation” as top nodes (i.e. with values of 0.54 and 0.52), but terms such as “cancer”, “gluten”, “pain”, “depression” and “probiotic” followed close behind (i.e. with values ranging between 0.50 and 0.48). This observation was somewhat expected considering that this is a highly connected network (i.e. the average number of neighbors is 12.631 and the characteristic path length is 2.961). Still, these values are interesting to identify good message “broadcasters”.

References

1. Collins CR, Stephenson K. A circle packing algorithm. Comput Geom [Internet] Elsevier; 2003 Jul 1 [cited 2018 Dec 20];25(3):233–256. [doi: 10.1016/S0925-7721(02)00099-8]
