# Supplementary material for: Using Twitter to Understand the Human Bowel Disease Community: Exploratory Analysis of Key Topics
Source: J Med Internet Res. 2019 Aug 15;21(8):e12610. doi: 10.2196/12610 (PMC6711036; doi:10.2196/12610)
Supplement: Multimedia Appendix 2 [file jmir_v21i8e12610_app2.pdf]

Multimedia Appendix 2

Using Twitter to understand the human bowel disease community: an exploratory analysis of key topics

Martín Pérez-Pérez[1,3,4], Gael Pérez-Rodríguez[1,3,4], Florentino Fdez-Riverola[1,3,4], Anália Lourenço[1,2,3,4*]

[1]The Biomedical Research Centre, Campus Universitario Lagoas-Marcosende, 36310 Vigo, Spain

[2]Centre of Biological Engineering, University of Minho, Campus de Gualtar, 4710-057 Braga, Portugal

[3]Department of Computer Science, University of Vigo, School of Computer Engineering, Campus As Lagoas, 32004 Ourense, Spain

[4]Next Generation Computer Systems Group, Galicia Sur Health Research Institute, Galician Health Service - University of Vigo, Spain

Email addresses:

MPP: martiperez@uvigo.es

GPR: gaeperez@uvigo.es

FFR: riverola@uvigo.es

AL: analia@uvigo.es

[*] Corresponding author:

Anália Lourenço [Tlf.: +34 988 387013, Fax: +34 988 387001]
ESEI: Escuela Superior de Ingeniería Informática. Edificio Politécnico. Campus Universitario As Lagoas s/n, 32004 – Ourense – Spain

Multimedia Appendix 2

Figure 1 illustrates the term co-occurrence network that was constructed to better understand what was being communicated in BD-related tweets for users identified as patients. The network was structured using the Circle Pack layout in order to keep the nodes with the same semantic category together [1]. The size of the nodes is based on the node degree (i.e. bigger nodes represent terms that are mentioned in more tweets), while the edge size is calculated based on the strength of co-occurrence (i.e. thicker edges represent higher term-term occurrences). The node color represents the semantic category of the term, i.e., blue stands for treatments, red represents drugs, yellow indicates diseases, green stands for foods and orange represents symptoms, while the edge color stands for the sentiment associated by the terms, namely, red represents a negative sentiment, green indicates a positive sentiment and grey stands for a neutral sentiment.

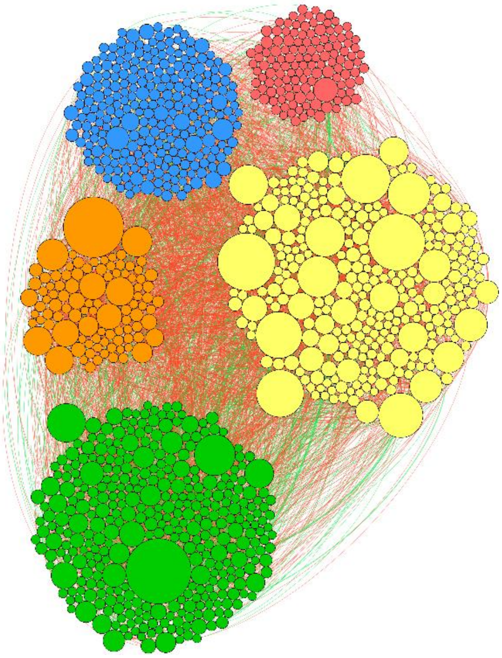

Figure 1. Network representing the co-occurrence of terms in bowel disease-related tweets for users identified as patients. The size of the nodes is based on the number of tweets mentioning the term while node color represents the corresponding semantic category. The edge color indicates the associated sentiment.

This network is composed of a total of 1,127 nodes and 6,085 edges, with an average number of neighbors of 10.799 and a characteristic path length of 3.045. Note that, explicit mentions to IBS and IBD (e.g. 'Inflammatory Bowel Disease' or 'Ulcerative Colitis') and non-content bearing, generalist terms (e.g. 'Disease' or 'Food'), were at the top of term mentions (i.e. highest degree values) but were not considered in the analysis. The network had an average clustering coefficient of 0.557, which indicates a moderate overall interconnection of the terms (i.e. a clustering coefficient of 1 would indicate that every term co-occurred with every other term). In particular, only a few terms were recurrently mentioned in the tweets and so, the clustering coefficient of most of the terms is zero or below ~0.33 (i.e. very few connectivities with other terms).

Similarly, the behavior of degree distribution in this co-occurrence network of patients is similar to the network showed in Material Appendix 1. So, terms referring to the BD-related conditions, like “cancer” (i.e. 175 neighbors), “diarrhea” (i.e. 173 neighbors), “anxiety disorder” (i.e. 145 neighbors), “depression” (i.e. 139 neighbors) and “arthritis” (i.e. 136 neighbors) were among the top most connected nodes. Regarding the other semantic categories, the most referred terms were as follows: “pain” (i.e. 186 neighbours), “fatigue” (i.e. 84 neighbours), and “bloating” (i.e. 80 neighbours) in symptoms; “low fodmap diet” (i.e. 34 neighbours), “diet” (i.e. 31 neighbours), and “colonoscopy” (i.e. 31 neighbours) in treatments; “medical cannabis” (i.e. 58 neighbours), “cannabidiol” (i.e. 12 neighbours) and “lubiprostone” (i.e. 12 neighbours) in drugs; and, “gluten” (i.e. 203 neighbours), “probiotic” (i.e. 117 neighbours), and “sugar” (i.e. 108 neighbours) in foods. That is, the terms presented in conversations of patients were typically used to denote general debate about the conditions and possible food and diets to treat them, rather than more specific topics of discussion. Betweenness and closeness centrality metrics supported this rationale. The three terms with the highest betweenness were “gluten”, “diarrhea” and “pain” (i.e. with a value of 0.12, 0.10 and 0.09, respectively). Similarly, “cancer”, “pain” and “gluten” had the highest closeness values (i.e. with values of ~0.50). The term pairs “probiotic” and “yeast” (i.e. related in 0.8% of tweets), “constipation” and “diarrhea” (i.e. related in 0.6% of tweets), and “depression” and “anxiety disorder” (i.e. related in 0.6% of tweets) were the most frequent pairs.

References

1. Collins CR, Stephenson K. A circle packing algorithm. Comput Geom [Internet] Elsevier; 2003 Jul 1 [cited 2018 Dec 20];25(3):233–256. [doi: 10.1016/S0925-7721(02)00099-8]
